# Inositol Pyrophosphate-Controlled Kinetochore Architecture and Mitotic Entry in *S. pombe*

**DOI:** 10.3390/jof8090933

**Published:** 2022-09-02

**Authors:** Natascha Andrea Kuenzel, Abel R. Alcázar-Román, Adolfo Saiardi, Simon M. Bartsch, Sarune Daunaraviciute, Dorothea Fiedler, Ursula Fleig

**Affiliations:** 1 Eukaryotic Microbiology, Institute of Functional Microbial Genomics, Heinrich-Heine-University, Universitätsstrasse 1, 40225 Düsseldorf, Germany; 2 Medical Research Council Laboratory for Molecular Cell Biology, University College London, Gower St., London WC1E 6BT, UK; 3 Leibniz Forschungsinstitut für Molekulare Pharmakologie, Robert-Rössle-Straße 10, 13125 Berlin, Germany; 4 Institut für Chemie, Humboldt-Universität zu Berlin, Brook-Taylor-Straße 2, 12489 Berlin, Germany

**Keywords:** inositol pyrophosphates, IP_8_, kinetochore, centromere, mitosis, Asp1, PPIP5K, CCAN, CENP-O, Mal2, Fta2, chromosome segregation, cell cycle, fission yeast, *Schizosaccharomyces pombe*

## Abstract

Inositol pyrophosphates (IPPs) comprise a specific class of signaling molecules that regulate central biological processes in eukaryotes. The conserved Vip1/PPIP5K family controls intracellular IP_8_ levels, the highest phosphorylated form of IPPs present in yeasts, as it has both inositol kinase and pyrophosphatase activities. Previous studies have shown that the fission yeast *S. pombe* Vip1/PPIP5K family member Asp1 impacts chromosome transmission fidelity via the modulation of spindle function. We now demonstrate that an IP_8_ analogue is targeted by endogenous Asp1 and that cellular IP_8_ is subject to cell cycle control. Mitotic entry requires Asp1 kinase function and IP_8_ levels are increased at the G2/M transition. In addition, the kinetochore, the conductor of chromosome segregation that is assembled on chromosomes is modulated by IP_8_. Members of the yeast CCAN kinetochore-subcomplex such as Mal2/CENP-O localize to the kinetochore depending on the intracellular IP_8_-level: higher than wild-type IP_8_ levels reduce Mal2 kinetochore targeting, while a reduction in IP_8_ has the opposite effect. As our perturbations of the inositol polyphosphate and IPP pathways demonstrate that kinetochore architecture depends solely on IP_8_ and not on other IPPs, we conclude that chromosome transmission fidelity is controlled by IP_8_ via an interplay between entry into mitosis, kinetochore architecture, and spindle dynamics.

## 1. Introduction

Inositol pyrophosphates (IPPs) are highly energetic molecules that are derived from *myo*-inositol carrying monophosphates and one or two diphosphate groups at defined positions of the inositol ring. IPPs are present in all eukaryotes and were categorized as signaling molecules due to their rapid turnover [1]. The functions of these molecules are very diverse, ranging from defense against pathogens in humans and plants, to mammalian organ development and fungal morphogenesis and pathogenicity [2,3,4,5,6,7,8]. Additionally, IPPs play an important function in cell adaptation to adverse environmental conditions, including a predominant role in maintaining phosphate homeostasis in mammals, plants, and fungi [4,9,10,11,12,13,14,15,16,17,18,19]. Although IPPs appear to have rather pleiotropic effects, evidence is accumulating that different phenotypes are connected, such as fungal virulence and phosphate homeostasis [20]. Importantly, the cellular levels of IPPs are responsive to different extrinsic signals [21,22] underscoring their role in regulating intracellular processes in response to environmental changes [23,24].

IPPs regulate cellular processes via distinct modes of action including direct binding to proteins and pyrophosphorylation of a protein at a pre-phosphorylated serine residue [16,18,20,25,26,27,28]. There are two highly conserved, exclusively eukaryotic enzyme families that generate the two IPPs, 5-PP-IP_5_ (IP_7_) and 1,5-(PP)_2_-IP_4_ (IP_8_) through the IP6K and Vip1/PPIP5K kinases, respectively [29,30,31,32,33,34].

**Figure 1:**
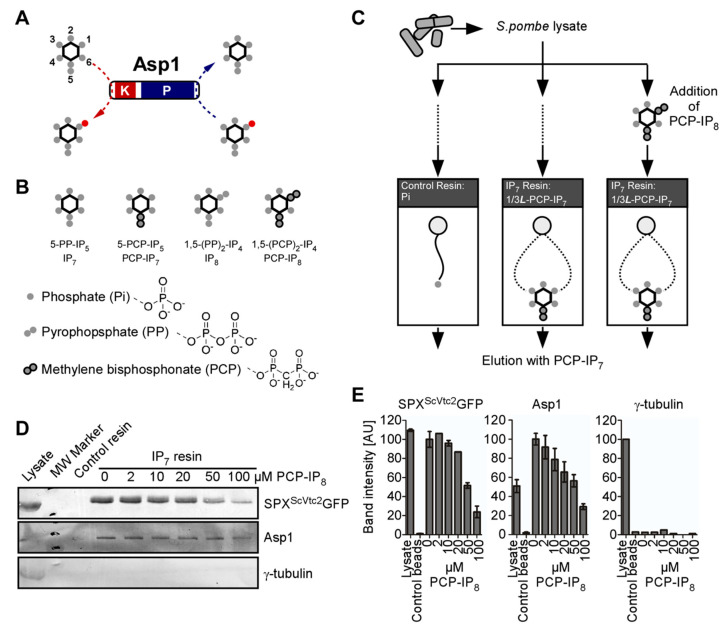
**Endogenous Asp1 targets IPPs.** (**A**) Schematic representation of the catalytic activities of *S. pombe* Asp1; K, kinase domain; P, pyrophosphatase domain. The *myo*-inositol ring is represented by a hexagon and the position of the phosphates (filled gray circles) on the inositol ring are numbered. The 1-β-phosphate (position C1), which is added and removed by Asp1, is colored red. (**B**) Graphical depiction of IP_7_ and IP_8_ and their corresponding metabolically-stable analogues. (**C**) Experimental overview of the affinity enrichment strategy and reagents that were utilized for the analysis of *S. pombe* cell lysates. The lysate was aliquoted and a subset of these aliquots was spiked with different concentrations of PCP-IP_8_ prior to incubation with control or IP_7_ resin. The linker that was utilized for the immobilization of Pi is depicted as a solid line. PCP-IP_7_ is immobilized on beads through a linker that is attached to the phosphate at either position C1 or C3 of the inositol ring (1/3***L***-PCP-IP_7_). All of the samples were eluted with PCP-IP_7_. (**D**) Western blot analysis of lysate and eluates from control and IP_7_ resin. The final concentration of PCP-IP_8_ that was used for each sample is noted. Endogenous proteins were detected with antibodies against Asp1 or γ-tubulin. A GFP antibody was used to detect SPX^ScVtc2^GFP. 1 technical replicate shown. (**E**) Band intensity quantification of proteins that were analyzed by Western blots in (**D**). Asp1 = 3; SPX^ScVtc2^GFP = 2; γ-tubulin = 1 technical replicate. Arbitrary units (AU).

As other members of the Vip1/PPIP5K family, the fission yeast *Schizosaccharomyces pombe* Asp1 protein is a bifunctional enzyme consisting of an N-terminal kinase domain with 1-kinase activity generating the less abundant IPP IP_8_ and a [2Fe-2S]-binding C-terminal pyrophosphatase domain with specific inositol pyrophosphate 1-phosphatase activity (Figure 1A [7,10,13,35,36,37]. Asp1 was originally discovered as a modulator of the cortical actin cytoskeleton, but since then numerous biological functions have been identified to be regulated by this protein [6,7,13,19,38,39,40,41]. In particular, the dynamics of the microtubule (MT) cytoskeleton in interphase cells, spindle assembly, and the dynamics and correct association of spindle MTs with the duplicated sister chromosomes are regulated by IP_8_ levels in a dose-dependent manner [39]. Cells that are unable to generate IP_8_ delay entry into anaphase A due to an activated spindle assembly checkpoint (SAC) indicating a defect in the correct binding of MTs to kinetochores, the attachment point on chromosomes, reviewed in [42]. Genetic deactivation of the SAC in IP_8_-deficient cells resulted in significant chromosome missegregation and the generation of aneuploid cells. Intriguingly, higher than wild-type IP_8_ levels had the opposite effect: chromosome biorientation, i.e., the correct association of sister chromosomes with MTs was faster than in the wild-type and increased chromosome transmission fidelity of a specific chromosome [39]. Thus, Asp1 kinase activity is required for genome stability and the avoidance of aneuploidy events, i.e., the loss or gain of whole chromosomes, which is a hallmark of cancer cells and found in several neurodegenerative diseases, reviewed in [43,44]. To better define Asp1-mediated transition through mitosis, we now determined if IP_8_ was required for entry into M-phase and which components of the MT-kinetochore interface other than MTs were subject to regulation by IP_8._ In particular, we analyzed if yeast strains expressing mutant components of the kinetochore were affected by varying IP_8_ levels. The huge macromolecular kinetochore complexes, which are several-fold larger than ribosomes are assembled on centromeric chromatin, which is defined in many organisms via the centromere-specific histone H3 variant, CENP-A [45], reviewed in [46,47]. Kinetochore composition is conserved and subcomplexes can be roughly classified as inner (close to the centromeric chromatin) and outer (close to/associating with spindle MTs) kinetochore subcomplexes. The 10-component outer subcomplex KMN (NMS in *S. pombe*) is the main platform for attachment of spindle MTs and the number of copies at the kinetochore and kinetochore localization per se are cell cycle-controlled in higher eucaryotes [48]. Interestingly, it has been shown recently, that this type of dynamic kinetochore composition is also present in *S. pombe* [49]. The role/importance/presence of specific components of the inner subcomplex constitutive centromere-associated network CCAN (in vertebrates/Ctf19 in *S. cerevisiae*/Mis6-Mal2-Sim4 in *S. pombe*) is somewhat variable depending on the organism that is being analyzed. For example, the *S. pombe* Mal2, the *S. cerevisiae* Mcm21, which is part of the COMA subcomplex and human CENP-O which belongs to CENP-OPQRU subcomplex are all members of one protein family. However, Mal2 is an essential protein, while Mcm21 is not and CENP-O requirement depends on the cell type [50,51,52]. Overall CCAN recruits and is required to maintain the specific histone H3 variant CENP-A, links centromeric chromatin and outer kinetochore, and recent cryo-EM structures of CCAN and Ctf19 demonstrated the importance of this subcomplex in dealing with spindle-generated forces, reviewed in [47,53,54,55,56,57]. Importantly, although the name CCAN implies that this complex is present throughout the cell cycle, the abundance of CCAN components can vary during the cell cycle [58].

Our present analysis of kinetochore targeting of fission yeast kinetochore proteins, identifies a new regulator of kinetochore architecture: intracellular IP_8_, which modulates kinetochore presence of specific *S. pombe* CCAN components in a dose-dependent manner.

## 2. Materials and Methods

### 2.1. Fission Yeast Strains and Plasmids

All of the strains are listed in Table S1. The plasmids that were used are listed in the Table S2. New strains were obtained by crossing the initial strains followed by random spore analysis or tetrad dissection. *S. pombe* strains were grown in rich (YE5S) or minimal medium (MM) with supplements [59]. To repress/de-repress the *nmt1*/*nmt41* promoter, the transformed cells were grown in MM with or without 5 µg/mL thiamine, respectively. This leads to low or high expression of the ORF of interest. For serial dilution patch tests, 10^4^ to 10^1^ cells of the indicated strains that were transformed with the relevant plasmid were grown under plasmid-selective conditions at the indicated temperatures.

For overexpression of *kcs1*^+^, the genomic DNA region encoding *S. pombe kcs1*^+^ was amplified from the wild-type yeast strain KG425 and ligated into pJR1-41XL [60] that was cut with Bsp68I using a blunt ligation approach giving rise to plasmid pUF1489. For the plasmid expressing SPX^ScVtc2^GFP (pUF1577), the genomic DNA fragment encoding the *Saccharomyces cerevisiae* Vtc2 protein, amino acids 1 to 146 (SPX domain) were amplified from a CEN.PK strain and ligated into the XhoI and NotI sites upstream of a nuclear localization signal that was fused to GFP that was previously cloned into plasmid pREP3XL [60] (pUF1027).

### 2.2. Affinity-Enrichment of IPP Binding Proteins

Harvesting and cryogenic lysis of *S. pombe* cells was performed adapting a previously described protocol for *S. cerevisiae* [61]. In short, wild-type fission yeast that was transformed with a plasmid encoding SPX^ScVtc2^GFP under the control of the *nmt1* promoter was grown in 2 l of MM with supplements without thiamine under plasmid selective conditions for 16 h at 25 °C. The culture was grown until an OD_600_ of 0.47 was reached and was then harvested by centrifugation at 3500× *g* for 10 min at 4 °C. The cells were collected and washed three times with ice-cold 50 mM Tris pH 7.5 with subsequent 5 min centrifugation at 2600× *g* at 4 °C. The buffer was removed and the pellet was centrifuged two more times removing as much of the buffer as possible after every spin. The yeast pellet was transferred to a syringe with a small spatula and slowly pressed with a plunger to generate short yeast noodles directly into a 50 mL falcon tube that was filled with liquid nitrogen. Liquid nitrogen was decanted and the noodles stored at −80 °C. The yeast noodles were transferred to a 35 mL Steel Mixer Mill Grinding Jar (Retsch) that had been previously cooled down with liquid nitrogen. A cooled 10 mm steel ball was placed on top of the noodles and the jar was closed. The jars were placed in a CryoMill (Retsch) and shaken for 3 min at a frequency of 30/s. The jar was cooled down with liquid nitrogen and then shaken again for a total of 5 cycles. The resulting frozen powder was stored at −80 °C. Lysis was checked microscopically with thawed powder.

The frozen yeast lysate powder was resuspended in ice-cold lysis buffer (50M Tris-HCl, pH 7.4, 150 mM NaCl, 0.05% Triton X-100, 1× Roche cOmplete Proteinase inhibitor cocktail) to a concentration of 6 µg/µL and rotated for 10 min at 4 °C before centrifugation at 3000× *g* for 10min at 4 °C. The lysate was aliquoted and specified samples were spiked with different concentrations of PCP-IP_8_ that were synthesized as in [62]. The lysates were then incubated with 100 µL equilibrated PCP-IP_7_ beads that were generated as in [63,64] with constant rotation at 4 °C. The samples were then centrifuged at 2000× *g* for 2 min and the supernatant was removed. The beads were washed three times with 1 mL lysis buffer for 5 min with rotation and centrifugation at 2000× *g* for 2 min. The beads were then incubated with 100 µL of lysis buffer containing 5 mM PCP-IP_7_ for 30 min under constant rotation at 4 °C. Finally, the samples were centrifuged and the supernatant was collected as the elution fraction, which was further analyzed by Western blotting using anti-GFP, anti-Asp1, and anti-γ-tubulin antibodies (Table S2). The quantification of the band intensity was performed with Image Lab (Bio-Rad, Hercules, CA, USA).

### 2.3. Microscopy

Immunofluorescence microscopy of fixed *S. pombe* cells was carried out as described [65]. The transformed cells were grown in plasmid-selective MM without thiamine overnight prior to analysis. First antibody used: monoclonal α-tubulin antibody TAT1 [66]. Secondary antibody: α-mouse Alexa Fluor^®^488 (1:200; Thermo Fisher Scientific, Waltham, MA, USA). DNA was stained with 4,6-diamidino-2-phenylindole (DAPI). The phenotypes were determined and counted visually using a Zeiss Axiovert200 fluorescence microscope. Pictures that are shown in Figure 3D were taken using a Nikon Eklipse Ti microscope.

**Figure 2:**
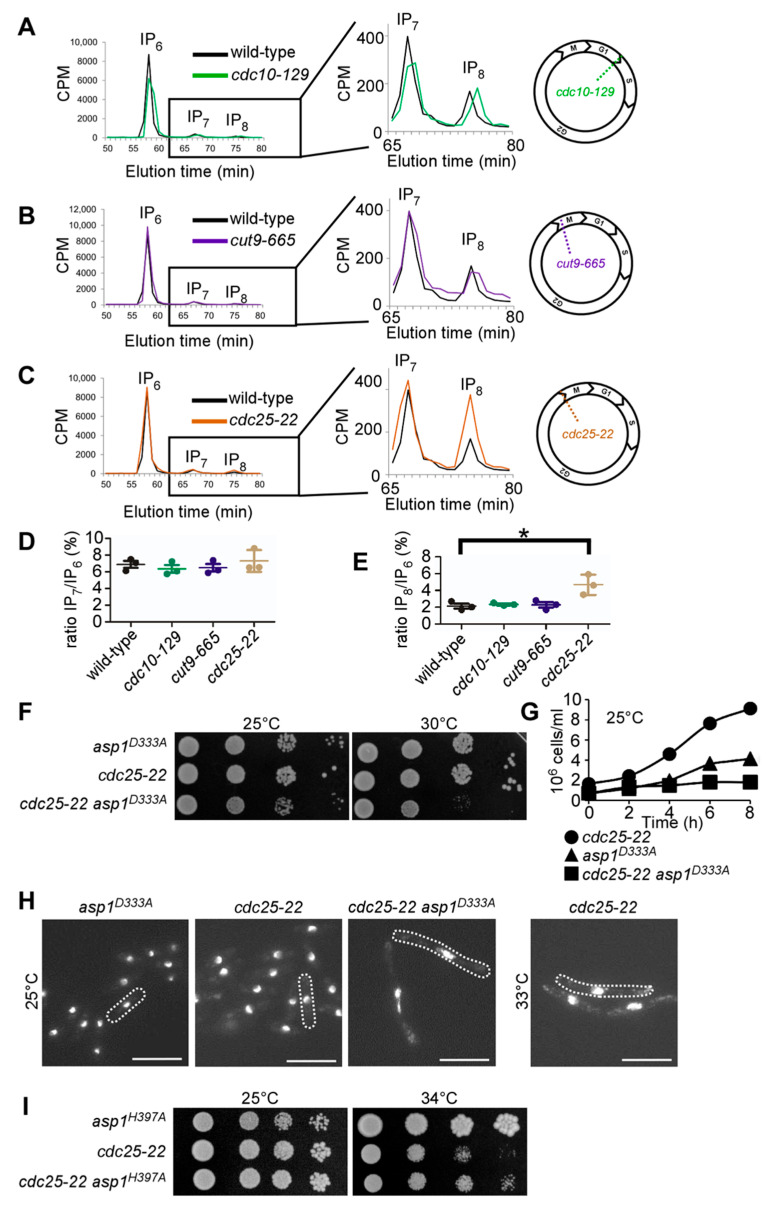
**IP_8_ is cell-cycle regulated and required for mitotic entry**. (**A**–**C**) Typical HPLC profiles of soluble inositol phosphates and pyrophosphates extracted from cells that were arrested in (**A**) G1 (*cdc10-129)*; (**B**) metaphase-anaphase A transition (*cut9-665)*, and (**C**) G2/M boundary (*cdc25-22)* compared to wild-type controls that were treated as the *cdc* strains. Boxed regions define the magnification of the IP_7_ and IP_8_ peaks shown on the right side. A schematic representation indicating where in the cell-cycle the indicated mutant arrests is shown in the right most panels. Wild-type and temperature-sensitive *cdc* strains were labeled with [^3^H] inositol and pre-grown at 25 °C before a shift to 36 °C for 6 h prior to the soluble inositol extraction. Counts per minute (CPM). (**D**) Quantification of the IP_7_ levels relative to IP_6_. Wild-type = 6.88 ± 0.72; *cdc10-129* = 6.35 ± 0.79; *cut9-665* = 6.48 ± 0.78; *cut25-22* = 7.29 ± 1.31 (**E**) Quantification of the IP_8_ levels relative to IP_6_. Wild-type = 2.12 ± 0.5; *cdc10-129* = 2.34 ± 0.14; *cut9-665* = 2.26 ± 0.57; *cdc25-22*: 4.68 ± 1.18. For (**D**,**E**), the mean and SD of n = 3 are shown. *, *p* = 0.0258 using a *t*-test. (**F**) Serial dilution patch test (10^4^–10^1^ cells) of indicated strains were grown for 3 (30 °C) or 4 days (25 °C) on YE5S plates. (**G**) Growth rates of the indicated strains grown in liquid YE5S at 25 °C. (**H**) Photomicrographs of fixed cells that were stained with DAPI to reveal the nucleus. Dotted lines indicate the cell periphery. Scale bars, 10 µm. (**I**) Serial dilution patch test (10^4^–10^1^ cells) of indicated strains grown for 4 days at the indicated temperatures on YE5S plates.

For DAPI staining of yeast cells, liquid cultures were grown at 25 °C overnight in YE5S. The cultures were then divided and grown for 6 h at either 25 °C or 33 °C. A total of 1 mL of each culture was centrifuged at 3000× *g* and fixed in 70% ice-cold ethanol. The cells were then washed 1× with PBS and stained with 100 ng/mL DAPI. Image acquisition for Figure 2H was done with a Zeiss Axiovert 200 fluorescence microscope (Carl Zeiss, Jena, Germany) using a 63× objective with a charge-coupled-device (CCD) camera (IEEE1394-Based Digital Camera Orca-ER 1394; Hamamatsu, Herrsching, Germany) and image editing and analysis was done with ImageJ 1.47v (National Institutes of Health).

For live-cell imaging, the cells were grown overnight at the indicated temperature in sterile-filtered MM with supplements (LFM: live fluorescence media). Microscopy slides were prepared by patching cells on agarose pads that were made of LFM containing 2% agarose and were sealed with VALAP (vaseline, paraffin and lanolin in a 1:1:1 ratio) [67]. A Zeiss spinning-disk confocal microscope that was equipped with a Rolera EM-C^2^ (QImaging) camera (or an Axiocam 702 mono camera or a Zeiss LSM 880 Airyscan microscope with GaAsP, PMT, and T-PMT detectors were used for imaging (CAi; HHU Düsseldorf). LSM: Figure 4B. Spinning disk: Figures 5C, 6B,C and Figure S3A,B. Imaging and analysis were performed with Zen2012 and AxioVision software. Image processing was done with ImageJ and Canvas 14. In all figures, clippings of the maximum intensity projection (MIP) images are shown. The contrast and brightness settings were chosen equally within the datasets.

### 2.4. Protein Extraction, IP, and Western Blot Analysis of Mal2-GFP

For Western Blot analysis of Mal2, strains were grown overnight in YE5S. A total of 5 × 10^8^–1 × 10^9^ cells were washed once with 5 mL STOP buffer (0.9% NaCl, 1 mM NaN_3_, 10 mM EDTA, and 50 mM NaF). The cells were resuspended in 500 μL HB15 lysis-buffer (25 mM MOPS, 60 mM ß-glycerophosphate, 15 mM p-nitrophenyl phosphate, 15 mM MgCl_2_, 15 mM EGTA, 1 mM DTT, 0.1 mM sodium orthovanadate, 1% TritonX100, 1 mM PMSF, and cOmplete protease inhibitor (Roche Diagnostics, Basel, Switzerland)) and lysed using glass beads [68]. The protein extract was cleared twice by centrifugation at 13,000 rpm for 30 min at 4 °C. For immunoprecipitation, 250 μL of each sample were incubated on ice for 1 h with 50 μL of α-GFP µMACS beads (Miltenyi Biotec, Bergisch Gladbach, Germany). The immunoprecipitates were isolated using the μMACS GFP isolation kit. The columns were equilibrated with 200 µL HB15 lysis-buffer before use. The columns were washed 8x with 200 µL HB15 lysis-buffer and the proteins were isolated in 2 × 50 µL elution buffer. The eluates or whole cell extracts were resolved on 10% SDS-gels before blotting. If the expected proteins were of different molecular weights, the membranes were cut to detect different proteins on one membrane. Antibodies that were used in Western blot analysis: monoclonal α-GFP (1:1000; mouse; Roche), α-GAPDH (1:3000; mouse; Sigma Aldrich, St. Louis, MO, USA), and α-γ-Tubulin (1:10,000; mouse; Sigma Aldrich).

To quantify the protein levels, ImageJ 1.44 (NIH) was used to measure the intensity of the protein bands in question. Protein amounts were normalized to the control signal for GAPDH or γ-Tubulin. The value was set to 1 for the wild-type (Figure 5B).

### 2.5. qChIP

Chromatin immunoprecipitation (ChIP) with *mal2*^+^-*gfp S. pombe* strains was performed as follows [69,70,71]. A total of 200 mL overnight cultures (sterile-filtered MM with supplements) with an OD_600_ of 0.4–0.8 that were grown at 25 °C from liquid pre-cultures were used. The cells were fixed with 3% paraformaldehyde for 30 min at 25 °C followed by washing twice with 20 mL cold 1× PBS and spheroplasted in 20 mL PEMS (100 mM Pipes, pH 7, 1 mM EDTA, 1 mM MgCl_2_, 1.2 M sorbitol) with 50 mg/mL Lallzyme at 37 °C for 30–45 min. The samples were washed twice in 10 mL PEMS and resuspended in 1 mL PEMS. The sample was divided onto two fresh tubes (no antibody control and IP sample). The cells were pelleted and the pellets were frozen at −20 °C until further use. The pellets were resuspended in 400 µL cold lysis buffer (50 mM HEPES-KOH pH7.5, 140 mM NaCl, 1 mM EDTA, 1% Triton-X-100, 0.1% sodium deoxycholate) that was supplemented with 1:100 cOmplete protease inhibitor cocktail and 2 mM PMSF (added shortly before use). The samples were sonicated twice for 6 sec at 10%. The supernatant was cleared via two 15,000 rpm centrifugation steps at 4 °C for 5 and 10 min. A total of 25 µL of protein A agarose was added to each sample and the tubes were incubated on a wheel at 4 °C for 1–2 h. The supernatant was cleared by centrifugation at 8000 rpm for 5 min at 4 °C. A total of 40 µL of each sample was frozen for later processing as input control. Then, 2 µL α-GFP antibody and 25 µL protein A agarose was added to the remaining lysates and precipitation was performed at 4 °C on a wheel overnight. The protein A agarose was pelleted via centrifugation at 8000 rpm for 5 min at 4 °C. Protein A agarose was washed with 1 mL of the following buffers followed by 5 min spinning on a wheel and 2 min centrifugation at 8000 rpm at each step: (1) lysis buffer, (2) lysis buffer with 0.5 M NaCl, (3) wash buffer (10 mM Tris/HCl pH: 8, 250 mM LiCl, 1 mM EDTA, 0.5% NP-40, 0.5% sodium deoxycholate), and (4) TE-buffer. A total of 250 µL TES (TE-buffer + 1% SDS) was added to the agarose and 210 µL to the formerly frozen 40 µL input control and the samples were incubated at 65 °C overnight in a water bath. The supernatant was cleared by 1 min centrifugation at 8000 rpm. A total of 450 µL TE that was supplemented with 30 µL freshly prepared Proteinase K (10 mg/mL) were added to each tube followed by incubation for 4 h at 37 °C while shaking. Afterwards phenol:chloroform and chloroform extraction were performed. DNA was precipitated by the addition of 1:10 *v/v* 3 M NaAc pH 5.5 and 2.5:1 *v*/*v* 96% EtOH and incubation on dry ice for 1 h. The samples were centrifuged for 30 min at 15,000 rpm at 4 °C and pellets were air-dried. The IP samples were resuspended in 30 µL TE-buffer and inputs in 300 µL TE-buffer. The samples were stored at −20 °C and 5 µL of 1:10–1:50 dilutions (equal within one experimental set and decided after analysis of the DNA amount in the input sample on an agarose gel) was used for qPCR. For qPCR reactions, the GoTaq qPCR Master Mix (Promega) was used following the manufacturers instructions. For each sample, qPCR was performed with two primer sets. The first set amplifies a region within the central region of centromeres 1 and 3 (cen1/3). The second set amplifies a fragment within the *act1*^+^ locus on chromosome 2 (actin) [72,73]. The oligonucleotides are listed in Table S2.

### 2.6. [^3^H]Inositol Labeling and HPLC Analysis

For [^3^H]inositol labeling and soluble inositol extraction, *S. pombe* cultures were grown overnight in MM containing all supplements and 10 μM inositol at 25 °C [36]. For cell-cycle arrest experiments, the cultures were then diluted to an OD_600_ of 0.05 in 5 mL MM that was supplemented with 10 μM inositol and 5 μCi/mL of [^3^H]inositol (10–25 Ci/mmol) and incubated at 25 °C. After 15 h, each culture was shifted to 36 °C for 6 h. For the non-shifted cultures, the cells were kept at 25 °C.

Inositol polyphosphates were extracted from the labeled cultures. The cells were centrifuged for 2 min at 2000 rpm at room temperature, washed once with 1 mL H_2_O, and transferred to 1.5 mL tubes. The cells were resuspended in 200 µL 1 M Perchloric acid + 5 mM EDTA (freshly added). ~1/2 PCR tube of glass beads were added and the cells were lysed in a gene disrupter for 5 min at 4 °C. The supernatant was cleared by 5 min centrifugation at 14,000 rpm at 4 °C. The cell pellet was kept at this step; later 1 mL 0.1% NaOH + 0.1% SDS were added to measure the total amount of radioactivity later; the samples were rotated on a wheel at room temperature overnight. A total of 45 µL of 1 M K_2_CO_3_ + 5 mM EDTA (freshly added) were added to the supernatants and the tubes were kept on ice for 2 h with their lids open (tubes were flicked gently every 15–30 min; CO_2_ bubbles form and evaporate). The pH of the samples was determined and had to be between 6 and 8. The samples were centrifuged for 5 min at 14,000 rpm at 4 °C and supernatants were transferred to a new tube and kept at 4 °C until HPLC analysis. Inositol polyphosphates were then resolved by strong anion-exchange SAX-HPLC (using a PartiSphere SAX 4.6 × 125 mm column; Hichrom). 1 mL fractions were collected each minute over 80 min. Then, 4 mL of Ultima-Flo AP scintillation cocktail was added to each fraction followed by vigorous mixing. All of the fractions were analyzed via scintillation counting [74].

### 2.7. Quantification of IP_8_/IP_6_ Ratios

The CPM values that were measured were used to generate the graphs that are shown in Figure 2. To calculate the IPP ratios, we first determined the background signal by measuring CPM in the last two fractions of the entire HPLC run. This amount was subtracted from all the values that were used for the quantification of peaks. The values that were part of the peaks for IP_6_, IP_7_, and IP_8_ were summed up for calculation. The ratio is given as percentage of the indicated IPP when compared to IP_6_.

## 3. Results

### 3.1. IP_8_ Is Targeted by Endogenous Asp1 and Its Level Is Cell-Cycle Controlled

We and others have described the catalytic properties of recombinant Asp1 purified from bacterial expression systems [7,10,31,36,75]. In these in vitro assays, Asp1 kinase and pyrophosphatase domains can independently interact with IPP substrates and perform their catalytic functions at position C1 of the inositol ring before releasing their products (shown diagrammatically in Figure 1A). To determine the interaction of endogenous wild-type Asp1 protein with its IP_7_ substrate, we performed an affinity-based enrichment utilizing the metabolically stable IP_7_ analog 5-PCP-IP_5_ (PCP-IP_7_) (Figure 1B) immobilized to agarose beads (IP_7_ resin). As control beads, the same beads and linker attached to a negatively charged phosphate group were used (control resin) (Figure 1C). As a positive control for IP_7_ binding, we used the SPX domain of the *S. cerevisiae* Vtc2 protein, a well-characterized IPP-binding module [16], fused to GFP (SPX^ScVtc2^-GFP). Additionally, in order to observe if IP_8_ was able to compete for binding to IP_7_, part of the *S. pombe* lysate was first incubated with different amounts of the IP_8_ analogue 1,5-(PCP)_2_-IP_4_ (PCP-IP_8_) before exposing it to the IP_7_ resin.

Clarified lysates of a wild-type fission yeast strain (i.e., expressing endogenous *asp1*^+^), transformed with a plasmid encoding SPX^ScVtc2^-GFP were spiked with 0, 2, 10, 20, 50, or 100 µM of PCP-IP_8_ and applied to the control or IP_7_ resin, followed by washes with binding buffer and elution with 10 mM PCP-IP_7_. The eluates were analyzed by Western blotting using GFP, Asp1, and γ-tubulin antibodies (Figure 1D,E). Both SPX^ScVtc2^-GFP and Asp1 bound to the IP_7_ resin with great specificity when compared to the control beads, similar to what has been found for the human PPIP5K family members [64]. However, pre-incubation of lysates with PCP-IP_8_ hindered the binding of SPX^ScVtc2^-GFP to the IP_7_ resin, confirming that SPX domains can bind IP_8_ [76]. Interestingly, binding of the Asp1 protein to IP_7_ resin was blocked by PCP-IP_8_ presence in a dose-dependent manner. This observation demonstrates that neither the kinase nor the phosphatase domain of the endogenous Asp1 bifunctional protein can bind to IP_7_ in the presence of high concentrations of IP_8_, likely due to direct competition of the IPPs for both of Asp1′s active sites.

To decipher the numerous biological roles of *S. pombe* Asp1, we previously used strains expressing Asp1 mutant variants which gave rise to altered intracellular IPP levels [6,7,36,39]. This was accomplished by mutations in either the kinase—or pyrophosphatase domain of the Asp1 protein [36] and this decrease/increase of the IP_8_ output had consequences for cellular functions that were regulated by IP_8_ in fission yeast in a dose-dependent manner [6,7,36,39]. However, it was unclear if physiological modulations of this bifunctional enzyme activity occur especially as Asp1 protein levels appear to remain constant during the cell cycle [77]. Thus, we assayed if the IP_8_ levels changed during the *S. pombe* cell-cycle. The inositol polyphosphate species were determined in three temperature-sensitive cell cycle mutant strains, *cdc10-129*, *cdc25-22*, and *cut9-665*, which arrest at the restrictive temperature in G1, at the G2/M transition and before anaphase A, respectively [78,79,80,81]. The mutant cells were pre-grown at the permissive temperature of 25 °C followed by 6 h incubation at the restrictive temperature of 36 °C. The cell cycle arrest was monitored by microscopy as arrested *cdc10-129* and *cdc25-22* cells are elongated and the arrested *cut9-665* cells show highly condensed chromosomes. To quantify the abundance of IP_7_ and IP_8_ in these strains, its precursor IP_6_ was used as a reference. The ratio of IP_7_/IP_6_ was not influenced by the cell-cycle stage in any of the three mutant strains that were tested (Figure 2A–C; quantification in Figure 2D). However, while the IP_8_/IP_6_-ratios in cells that were arrested in G1 (*cdc10-129*) and at the metaphase/anaphase A transition (*cut9-665*) were comparable to the non-arrested wild-type cells that were incubated at 36 °C (Figure 2E), the IP_8_/IP_6-_ratio was significantly increased in the *cdc25-22* cells (Figure 2C and quantification in Figure 2E).

Mitosis is universally initiated by the activation of Cdk1 protein kinases, which in turn are controlled by Cdc25 phosphatases [82]. Thus, *cdc25-22* mutant cells arrest in late G2 at the transition to M-phase and at this cell cycle stage the IP_8_/IP_6-_ratio was increased. Such an increase was not observed at the permissive temperature of 25 °C, when these cells can progress through the cell cycle (Figure S1A–C). As the increase at the G2/M transition might indicate a particular relevance of IP_8_ at this cell cycle stage, we tested if IP_8_ was required for mitotic entry. We, therefore, generated a *cdc25-22 asp1^D333A^* double mutant strain. The Asp1^D333A^ mutant protein carries a single amino acid change in the catalytic domain of the kinase which leads to a kinase-dead version and no cellular IP_8_ [36] (Figure 5A). Patch test analysis of the double mutant and parental strains showed that the *cdc25-22 asp1^D333A^* strain grew slightly slower on solid media at 25 °C than the single mutant *asp1^D333A^* strain and had a clearly reduced growth at 30°C compared to the parental strains (Figure 2F). In addition, the generation time of the double mutant at 25 °C was nearly 6 h, twice as long as that of the parental strains, which resulted in a very shallow growth curve in liquid media when compared to the single mutant strains (Figure 2G). Microscopic analysis of double mutant cells grown at 25 °C in liquid revealed that these cells were highly elongated with a stretched-out nucleus (Figure 2H). This phenotype is the hallmark of *cdc25-22* cells that are incubated at semi-permissive/restrictive temperatures (Figure 2H, right most panel) [83]. Intriguingly, when *cdc25-22* cells have more IP_8_, i.e., in an *asp1^H397A^* background (Figure 5A diagrammatically depicts IPP levels in *asp1^H397A^* strain) the reduced growth phenotype that was seen for the *cdc25-22* mutant grown at the semi-permissive temperature of 34 °C is rescued (Figure 2I). We conclude that IP_8_ is required for entry into mitosis.

### 3.2. Alteration of Intracellular IP_8_ Levels Can Rescue Non-Growth of S. pombe CCAN Kinetochore Mutant Strains

**Figure 3:**
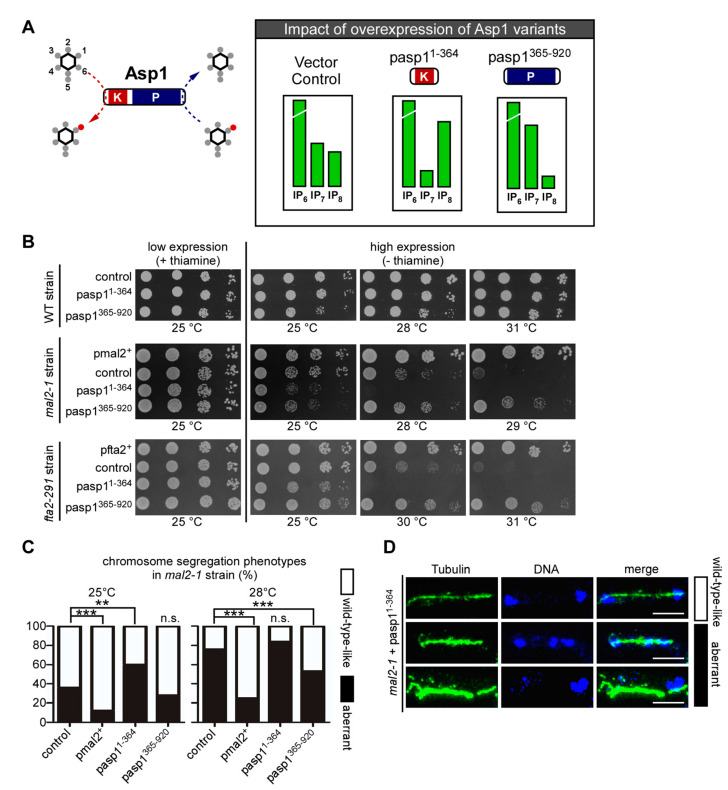
**Alteration of IP_8_ levels affect the temperature-sensitive growth phenotype of CCAN kinetochore mutant strains**. (**A**) Schematic representation of the impact in IP_7_ and IP_8_ levels upon the expression of the Asp1 kinase (Asp1^1−364^) and pyrophosphatase (Asp1^365−920^) variants [36,37]. (**B**) Serial dilution patch test (10^4^–10^1^ cells) of wild-type (WT), *mal2-1* and *fta2-291* strains that were transformed with the indicated plasmids and grown at the indicated temperature for 4–8 days depending on the incubation temperature. pmal2^+^; plasmid with wild-type *mal2*^+^ ORF expressed via the *nmt1* promoter. *nmt1* transcribed genes show low expression in the presence of thiamine and high expression when no thiamine is present. pasp1^1−364^ and pasp1^365−920^, plasmids with *nmt1* driven expression of the Asp1 kinase or pyrophosphatase domains, respectively. One of n = 3 shown. (**C**) Quantification of chromosome segregation phenotypes that were observed in a *mal2-1* strain transformed with the indicated plasmids. 25 °C: **, *p* = 0.0011 ***; *p* < 0.0001. 28 °C: ***, *p* < 0.0001 (*mal2*^+^); ***, *p* = 0.0007 (asp1^365−920^), n.s., not significant. Fisher’s exact test. n = 100 cells per plasmid. One of n = 2 shown. (**D**) Immunofluorescence of fixed *mal2-1* cells that were transformed with pasp1^1−364^ and grown at 25 °C. Green, α-TAT1 (tubulin); blue, DAPI staining. Scale bars, 5 µm.

Our finding that Asp1 kinase activity was required for entry into mitosis is in accordance with previous work where we have shown that chromosome segregation fidelity is modulated by Asp1 kinase function [39]. Specifically, we established that bipolar spindle formation/function and the accuracy of chromosome transmission required intracellular IP_8_ in a dose-dependent manner. To determine if the modulation of spindle dynamics was the sole target of Asp1 regulation during mitosis, we now tested if the conductor of mitosis, the kinetochore, was also subject to control by Asp1-made IPPs. We assayed if the expression of either the Asp1-kinase (Asp1^1−364^) or the Asp1-pyrophosphatase (Asp1^365−920^) altered the growth of specific kinetochore mutant strains. As shown diagrammatically in Figure 3A, plasmid-borne expression of *asp1^1−364^* increases intracellular IP_8_, while *asp1^365−920^* expression reduces (but does not eliminate) IP_8_ levels [36]. 

*S. pombe* kinetochores complexes consist of the same core modules found in human and *S. cerevisiae* such as KMN(human)/NMS(*S. pombe*) and CCAN(human)/Ctf19(*S. cerevisiae*)/Mis6-Mal2-Sim4(*S. pombe*) [84,85,86,87,88]. The expression of either *asp1^1−364^* or *asp1^365−920^* from a plasmid had no or only a moderate effect on the growth of four temperature-sensitive *S. pombe* KMN mutant strains that were tested (Figure S2A). However, when these Asp1 variants were expressed in four strains with mutant *S. pombe* CCAN components, we observed a strong effect between growth at higher temperatures and intracellular IP_8_ levels for three mutant strains (Figure 3B and Figure S2A,B). For example, the *mal2-1* strain was unable to grow at the semi-permissive temperature of 28 °C when *asp1^1−364^* expression resulted in higher intracellular IP_8_, while its non-growth phenotype at 29 °C was suppressed by the expression of *asp1^365−920^* (lower than wild-type IP_8_ level) (Figure 3B, middle panels). Similarly, the temperature-sensitivity of the *fta2-291* and *mis6-302* mutant strains was increased in cells with higher than wild-type levels of IP_8_ and decreased in cells with reduced IP_8_ level (Figure 3B, bottom panels; Figure S2B). Thus, the temperature-sensitive growth phenotype of mutant *S. pombe* CCAN components can be altered by varying intracellular IP_8_ levels.

To determine the molecular basis of this phenotype, we used the *mal2-1* mutant strain for further analysis. We assayed chromosome segregation in *mal2-1* transformants that were grown at 25 or 28 °C by microscopic analysis of fixed mitotic cells. Although *mal2-1* cells are able to grow at 25 °C, the fidelity of chromosome transmission is already decreased at this temperature and 36% of mitotic cells show unequal segregation of chromosomes due to non-disjunction of sister chromosomes as analyzed by staining fixed cells with DAPI and anti-tubulin antibody [50,70] (Figure 3C, examples of this phenotype are shown in Figure 3D). The aberrant phenotype at 25 °C of unequally/partially segregated chromatin on an elongating spindle was reduced to 12% and 28% in mitotic cell populations expressing plasmid-encoded wild-type *mal2*^+^ or *asp1^365−920^*, respectively. The expression of *asp1^1−364^* significantly increased the chromosome mis-segregation phenotype at 25 ° C to 60% (Figure 3C). A similar pattern was observed for transformed *mal2-1* cells that were incubated at 28 °C (Figure 3C), revealing an inverse relationship between chromosome segregation fidelity of *mal2-1* cells and IP_8_ levels.

### 3.3. Kinetochore-Targeting of the Mutant Mal2-1 Protein Is Subject to IP_8_ Levels

**Figure 4:**
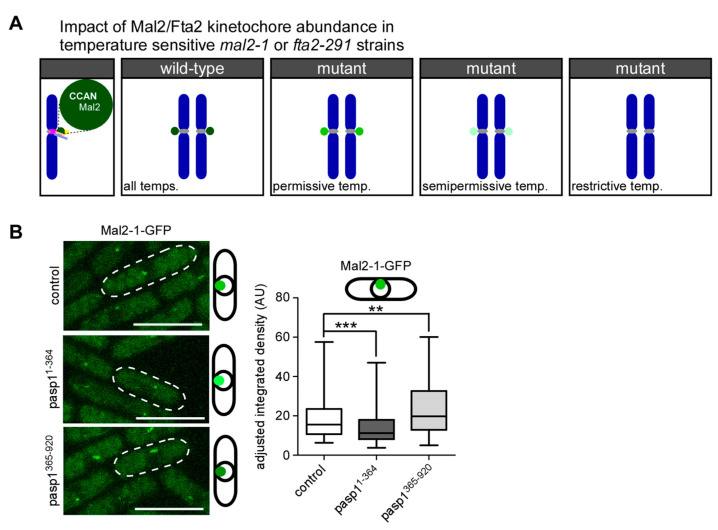
**Negative correlation between IP_8_ levels and Mal2-1-GFP kinetochore fluorescence**. (**A**) Diagrammatic representation to show that Mal2-1-GFP kinetochore targeting is temperature-dependent [91]. The lighter the shade of green, the less Mal2-1-GFP is at the kinetochore. (**B**) Left: Live cell images of the indicated *mal2-1-gfp* transformants grown at 25 °C. Scale bar, 10 µm. Right: Quantification of Mal2-1-GFP fluorescence signals: Mean and SD: vector = 18.42 AU ± 9.7; *asp1^1−364^* = 14.53 AU ± 6.39; *asp1^365−920^* = 22.77 AU ± 12.17. Number of kinetochore signals that were counted: control: = 162; *asp1^1−364^* = 160; *asp1^365−920^* = 140; ***, *p* < 0.0001; **, *p* = 0.0019; Mann–Whitney U-Test. One of n = 2 sets shown.

To analyze if kinetochore-targeting of Mal2-1-GFP was dependent on cellular IP_8_ levels, we performed live-cell fluorescence microscopy of endogenous *mal2-1-gfp* interphase cells that were transformed with plasmids expressing either *asp1^1−364^* or *asp1^365−920^*. The growth behavior of this strain transformed with pasp1^1−364^ or pasp1^365−920^ was comparable to the non-tagged *mal2-1* strain (Figure S2C). In interphase cells, the centromeres are clustered at the spindle pole body due to a linker complex and thus fluorescent kinetochore proteins are seen as a single dot-like signal in the nucleus [89,90]. The Mal2-1-GFP protein is present at the kinetochore at 25 °C, albeit in reduced amounts compared to wild-type Mal2-GFP. With increasing temperatures, the Mal2-1-GFP signal is further reduced or absent (Figure 4A) [50,70,91]. The expression of *asp1^1−364^* led to a significant reduction of the Mal2-1-GFP kinetochore signal, while the expression of *asp1^365−920^* increased kinetochore-presence of Mal2-1-GFP (Figure 4B). Thus, kinetochore targeting of the mutant Mal2-1 protein is modulated by the intracellular IP_8_ levels.

### 3.4. Wild-Type Mal2 Kinetochore-Targeting Is Modulated by Intracellular IP_8_ Levels

**Figure 5:**
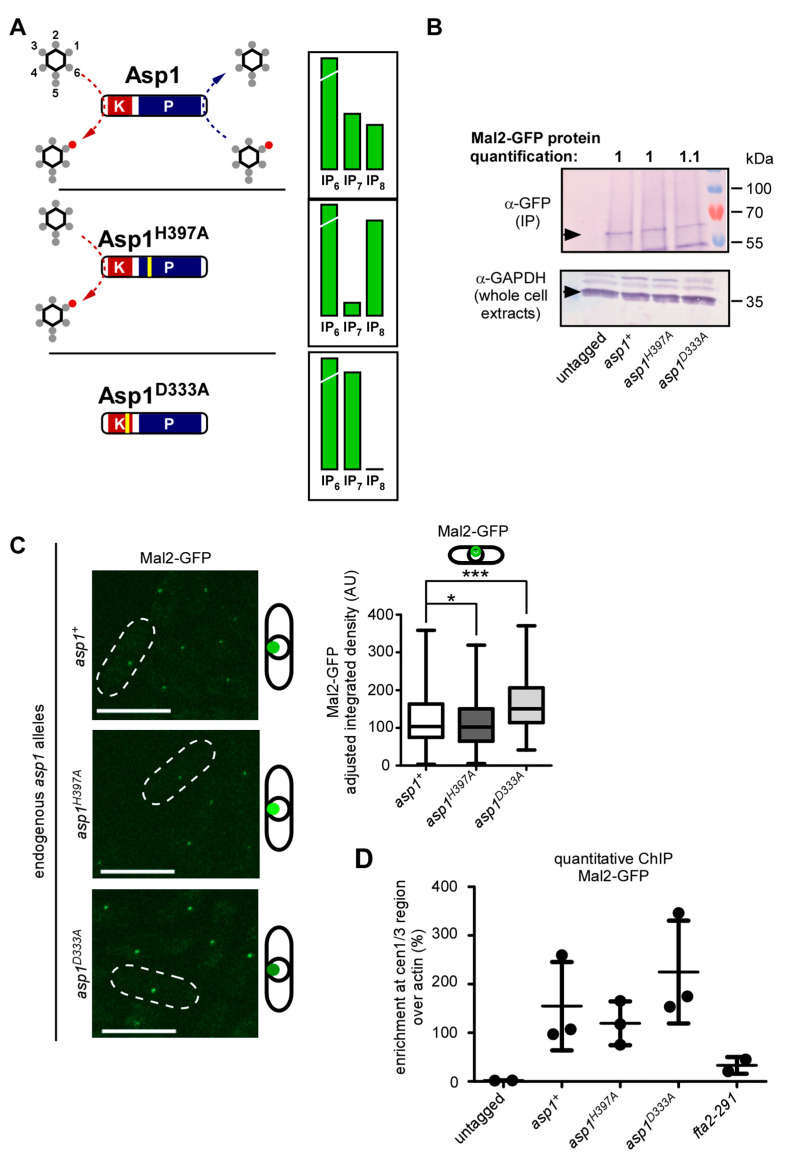
**Kinetochore-targeting of wild-type Mal2 is subject to IP_8_ levels**. (**A**) Schematic representation of the three endogenous Asp1 variants and their impact on IP_7_ and IP_8_ levels [36]. Note that in the case of Asp1^D333A^, no IP_8_ is generated and thus, no IP_8_ is being hydrolyzed, although the pyrophosphatase domain is intact. (**B**) Western blot analysis of Mal2-GFP protein levels in specified *asp1*-variant strains utilizing indicated antibodies. To detect Mal2-GFP, an immunoprecipitation with anti-GFP antibodies was performed prior to Western blot analysis. Quantification of the fold change of Mal2-GFP bands that were normalized to GAPDH are shown above the blot. One of n = 2 sets shown. (**C**) Left: Live-cell images of the indicated *asp1*-variant strains endogenously expressing *mal2*^+^-*gfp* grown at 25 °C. Scale bars, 10 µm. Right: Quantification of the Mal2-GFP fluorescence signals in each strain. Mean and SD: *asp1*^+^= 122.5 AU ± 63.67; *asp1^H397A^* = 110.8 AU ± 58.43; *asp1^D333A^*= 163.8 AU ± 65.53. The number of kinetochore signals that were counted, *asp1*^+^ n = 381; *asp1^H397A^* n = 381; *asp1^D333A^* n = 380. *, *p* = 0.0195; ***, *p* < 0.0001; Mann–Whitney U-Test. Average of n = 3 shown. (**D**) qChIP analysis for the strains that were imaged in (**C**). Shown is the enrichment of cen1/3 DNA relative to the *act1*^+^ locus. The mean and SD shown: untagged (wild-type strain) = 1.99 ± 0.32; *asp1*^+^ = 154.67 ± 90.99; *asp1^H397A^* = 119.55 ± 45.01; *asp1^D333A^* = 224.83 ± 105.56; *fta2-291* = 33.03 ± 17.06. Untagged and *fta2-291* strains, n = 2. *asp1*^+^, *asp1^D333A^*, *asp1^H397A^* strains n = 3 biological replicates with 2-4 technical replicates each.

To assess, if IP_8_ kinetochore-targeting also applied to the wild-type Mal2 protein, we determined endogenous Mal2-GFP localization in cells with twice as much IP_8_ as a wild-type strain (Asp1^H397A^) and cells with no IP_8_ (Asp1^D333A^), and compared them to a wild-type control (diagrammatically shown in Figure 5A; based on data from [36]. The total Mal2-GFP protein levels were unaffected by changes in the intracellular IP_8_ levels (Figure 5B). However, as had been observed for the mutant Mal2-1 protein, we found that Mal2-GFP kinetochore targeting was also controlled by IP_8_ levels (Figure 5C). Higher than wild-type intracellular IP_8_ significantly reduced Mal2-GFP kinetochore signal, while the absence of IP_8_ had the opposite effect (Figure 5C).

Next, we performed a quantitative chromatin immunoprecipitation (qChIP), in which Mal2-GFP was cross-linked to centromeric DNA and the abundance of these DNA regions (cen1/3) over a non-centromere region (actin) was quantified as a measure of kinetochore localization [69,70,71,73,92]. The mutant *fta2-291* strain was used as a negative control for Mal2-GFP kinetochore localization as Mal2 and Fta2 kinetochore localization is interdependent and their orthologs exist as a heterodimer [58,91,93,94,95]. As described previously, the presence of the mutant Fta2-291 protein led to massively reduced Mal2 at the kinetochore (Figure 5D; [91]). Compared to cells with physiological IP_8_ levels (*asp1*^+^ strain), the enrichment of cen1/3 over actin increased by ~45% in cells without IP_8_ (*asp1^D333A^* strain) and decreased by ~20% in cells with more than wild-type IP_8_ (*asp1^H397A^* strain) (Figure 5D). Thus, kinetochore-targeting of the wild-type Mal2 protein is dependent on the intracellular IP_8_ levels.

Finally, as the ts phenotype of the *fta2-291* strain was affected in a very similar way as the *mal2-1* strain by altered IP_8_ levels (Figure 3B), we determined if kinetochore targeting of the wild-type Fta2 protein was affected by IP_8_ levels. Fta2-GFP kinetochore-targeting was also IP_8_ regulated as it significantly increased in IP_8_-less cells and decreased in cells with higher-than-wild-type IP_8_ (Figure S3A).

The above microscopic analysis of kinetochore localization of Mal2-1-GFP, Mal2-GFP, and Fta2-GFP was carried out using heterogenous cell populations of interphase cells. To determine if altered IP_8_ levels also affected targeting of kinetochore proteins in M-phase, we analyzed Mal2-GFP kinetochore fluorescence in late anaphase cells and found that in cells without IP_8_ the Mal2-GFP kinetochore signal was also increased significantly (Figure S3B).

### 3.5. IP_8_, and Not IP_7_, Controls Kinetochore Targeting of S. pombe CCAN Components

Decreasing or eliminating IP_8_ through heterologous expression of the Asp1 pyrophosphatase variant Asp1^365−920^ or via the expression of endogenous *asp1^D333A^* (kinase-dead) resulted in an increase of the *S. pombe* CCAN members Mal2 and Fta2 at the kinetochore. However, one important consequence of decreasing IP_8_ levels in this fashion is a concomitant increase in IP_7_ (Figure 3A and Figure 5A). Thus, we needed to determine if the increase in IP_7_ levels might have a role in kinetochore targeting of *S. pombe* CCAN components.

**Figure 6:**
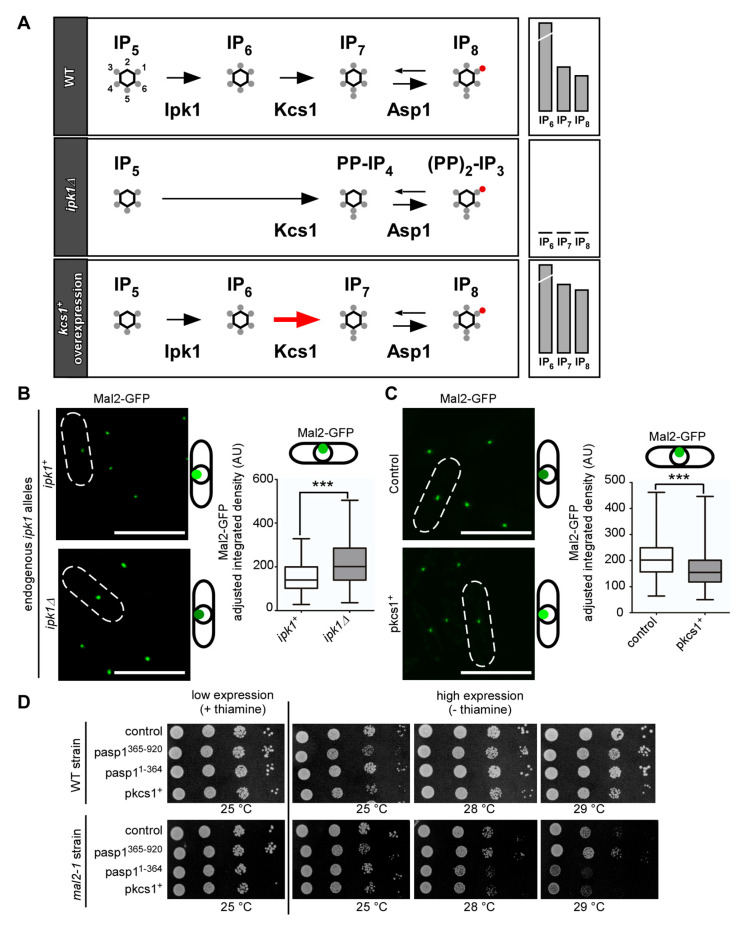
**Alterations of the IP_8_ synthesis pathway reveal that kinetochore targeting depends on IP_8_ and not IP_7_ levels.** (**A**) Schematic representation of part of the wild-type IP/IPP pathway from IP_5_ to IP_8_, and the changes in IP_7_ and IP_8_ in a *S. pombe ipk1*∆ strain [96] and a wild-type *S. cerevisiae* strain overexpressing *KCS1* (red arrow) based on [99]. (**B**) Left: Live-cell images of *ipk1*^+^ and *ipk1*∆ strains endogenously expressing *mal2*^+^-*gfp*. Cells were grown at 30 °C. Scale bars, 10 µm. Right: Quantification of Mal2-GFP kinetochore fluorescence signals in each strain. Mean and SD: *ipk1*^+^ = 152.3 AU ± 65.86; *ipk1*∆ = 213.1 AU ± 93.54. Number of kinetochore signals that were counted; *ipk1*^+^ = 243; *ipk1*∆ = 183. ***, *p* < 0.0001; Mann–Whitney U-Test. N = 2. (**C**) Left: Live-cell images of a wild-type strain that were transformed with either control plasmid or a plasmid harboring *kcs1*^+^ overexpressed via the *nmt41* promoter. The cells were grown at 30 °C. Scale bars, 10 µm. Right: Quantification of Mal2-GFP fluorescence signals in each condition. Mean and SD: control plasmid = 205.5 AU ± 65.8; *pkcs1*^+^ = 164.6 AU ± 63.39. Number of kinetochore signals that were counted; control n = 594; pkcs1^+^ n = 609; ***, *p* < 0.0001; Mann–Whitney U-Test. N = 2. (**D**) Serial dilution patch test (10^4^–10^1^ cells) of wild-type and *mal2-1* strains that were transformed with the indicated plasmids and grown at 25 to 29 °C and with/without thiamine for 4–8 days. One of n = 2–3 sets shown.

To answer this question, we utilized a genetic strategy. First, we eliminated cellular IP_6_, the fully mono-phosphorylated form of *myo*-inositol that serves as the precursor of yeast IPPs. IP_6_ is generated by Ipk1-mediated phosphorylation at position C2 of the inositol ring (diagram in Figure 6A, top panel). Cells which have the *ipk1*^+^ gene deleted, are unable to generate IP_6_, IP_7_, and IP_8_, and instead accumulate large amounts of the Ipk1 substrate IP_5_, along with unconventional IPPs that are not detected in wild-type *S. pombe* cells (Figure 6A, middle panel) [96]. In an *ipk1*∆ strain, the Mal2-GFP kinetochore signal at the kinetochore was significantly increased (Figure 6B), similar to what we observed for the *asp1^D333A^* strain (Figure 5C). Thus, in both the *ipk1*∆ and the *asp1^D333A^* strains, which have very different IP_7_ levels but share an inability to generate IP_8_, kinetochore targeting of Mal2-GFP was increased.

Next, we targeted the next enzyme in the IPP biosynthetic pathway, Kcs1, which uses IP_6_ as a substrate to generate IP_7_ (Figure 6A, top panel). In contrast to *S. cerevisiae*, where the deletion of *KCS1* is viable [97], *S. pombe kcs1*^+^ is an essential gene [98], (our analysis). Thus, we perturbed IP_7_ levels by the overexpression of *kcs1*^+^ on a plasmid via the *nmt41* promoter. Plasmid-borne overexpression of *KCS1* in budding yeast has been shown to increase both IP_7_ and IP_8_ levels [99] (diagrammatically shown in Figure 6A, bottom panel). If increased IP_7_ levels give rise to higher levels of Mal2 kinetochore targeting, we would expect that the overexpression of *kcs1*^+^ phenocopies the result that is observed for the *asp1^D333A^* strain (i.e., no Asp1 kinase activity increases intracellular IP_7_ levels compared to a wild-type strain). Instead, Mal2-GFP kinetochore targeting was decreased in cells overexpressing *kcs1*^+^ (Figure 6C), likely due to higher than wild-type IP_8_ levels in these cells. Furthermore, we analyzed the impact of *kcs1*^+^ overexpression on the growth of a *mal2-1* strain and found that it resulted in growth reduction at the semi-permissive temperature similar to *mal2-1* cells expressing *asp1^1−364^* on a plasmid (Figure 6D). As both types of yeast transformants have increased IP_8_ but opposite levels of IP_7_, we conclude that kinetochore targeting of *S. pombe* CCAN components is solely IP_8_-dependent.

## 4. Discussion

In this study we have demonstrated that Asp1 kinase function has a much broader impact on mitotic processes than previously determined: in a dose-dependent manner IP_8_ is required for entry into mitosis, spindle formation and function [39], and kinetochore architecture. The complete absence of IP_8_ led to defects in chromosome transmission fidelity resulting in aneuploidy and polyploidy [36,39]. Intriguingly, increasing IP_8_ beyond physiological levels improved transmission of a chromosome above that of a strain with wild-type IP_8_ levels, proposing that IP_8_ is an important player in chromosome transmission fidelity. Using kinetochore targeting of Mal2 as a tool, we have now also demonstrated that it is specifically the IPP IP_8_ that modulates mitosis.

In accordance with the importance of IP_8_ for entry and progression through mitosis, we found that IP_8_ levels are increased at the G2/M boundary but not in G1 or at the metaphase-anaphase A transition. Our analysis does not exclude that IPP levels are cell-cycle-regulated in other cell cycle phases apart from G2/M. Indeed, IPP levels change in *S. cerevisiae* cells during progression through S-phase [100]. How such an alteration of IPP levels is regulated in vivo, is unknown. Our finding that only IP_8_ but not IP_7_ levels are elevated at G2/M suggests that (i) it is specifically an IP_8_ increase that is required at this cell cycle stage and (ii) this alteration might be due to the downregulation of the Asp1-pyrophosphatase domain and not by other phosphatase activities [101,102,103,104].

IP_8_ is required for mitotic entry in a dose-dependent manner. At the permissive temperature for the single mutant *cdc25-22* strain, double mutant *cdc25-22 asp1^D333A^* (no IP_8_) cells showed all phenotypes indicative of a delay at the G2/M transition. The presence of more than the physiological levels of IP_8_ had the opposite effect and partially rescued the *cdc25-22* temperature-sensitive growth phenotype. How IP_8_ modulates mitotic entry on a molecular basis will be determined in future studies. However, it has been shown recently that the non-growth phenotype of strains with a deletion of *plo1*^+^, which encodes the essential *S. pombe* Polo-like kinase [105] can be rescued by two ways, both of which increase intracellular IP_8_ levels: mutations in the Asp1 pyrophosphatase domain or presence of non-functional Aps1, a member of the DIPP nudix phosphohydrolases that regulate IPP levels [7,103,106,107,108]. As extra IP_8_ is able to bypass the requirement for Polo kinase [106] and as this kinase regulates mitotic entry, in part by activating Cdc25 (reviewed in [109]), we speculate that IP_8_ does not modulate entry into mitosis via Cdc25.

Commitment to mitosis results in the formation of the bipolar spindle and the bioriented attachment of sister chromosomes via their kinetochores to MTs from the opposing spindle poles. We now demonstrate that kinetochore targeting of components of the fission yeast CCAN subcomplex [87] is subject to control by IP_8_ levels. The reduction of IP_8_ in temperature-sensitive CCAN mutant strains *mal2-1*, *fta2-291*, and *mis6-302* partially rescued the non-growth phenotype at higher temperatures, while increased intracellular IP_8_ had the opposite effect. Kinetochore targeting of Mal2-1, wild-type Mal2, and Fta2 in strains with different IP_8_ levels demonstrated an inverse relationship between IP_8_ levels and kinetochore association of these proteins. Furthermore, the reduction of IP_8_ increased kinetochore-localized Mal2 beyond that which is seen in wild-type cells suggesting that cellular pools of Mal2-GFP exceed the amount of proteins that are localized at the kinetochore; a characteristic that is also shown for human CENP-Q [58]. What could be the role of IP_8_-mediated kinetochore targeting of fission yeast CCAN components? A characteristic of the CCAN from yeast to humans is that it is present at the centromere during the entire cell cycle and CCAN subunits show a hierarchical set-up (reviewed in [110]). However, the abundance of individual components of the CCAN at the kinetochore can vary quite dramatically. For example, the levels of human CCAN members CENP-C/H/T in the kinetochore are 50% increased after nuclear envelope breakdown, while the CCAN member CENP-N exhibited the opposite behavior [111]. Artificial perturbations of IP_8_ levels by using Asp1 variants uncovered the ability of IPPs to modulate kinetochore architecture. However, the finding that IP_8_ levels change at the G2/M transition suggests that this also occurs under physiological conditions. We have assayed just a few members of the *S. pombe* CCAN for IP_8_-dependency but as CCAN components depend on each other for recruitment, it is possible, that changes in the CCAN via IP_8_ are substantial.

Kinetochore targeting of numerous kinetochore proteins is regulated by phosphorylation/dephosphorylation events [112], reviewed in [113]. As IPPs can modulate protein function via pyrophosphorylation of a pre-phosphorylated serine residue, we speculate that kinetochore targeting of *S. pombe* CCAN components could be mediated by pyrophosphorylation of a target protein/proteins.

## Data Availability

Data are contained within the article or supplementary material.

## Supplementary Materials

The following supporting information can be downloaded at: https://www.mdpi.com/article/10.3390/jof8090933/s1, Table S1: Strains used in this study; Table S2: List of reagents and plasmids; Supplementary Methods; Figure S1: IP_7_ and IP_8_ levels are similar in wild-type and *cdc25-22* strains grown at 25 °C; Figure S2: Growth phenotype of kinetochore mutant strains expressing *asp1^1−364^* or *asp1^365−920^* from a plasmid via the *nmt1* promoter; Figure S3: Effect of IP_8_ level changes on Fta2-GFP kinetochore targeting in interphase cells and Mal2-GFP late mitotic cells. References [114,115,116,117,118] are used only in the Supplementary Materials.
